# Intervention and research progress of gut microbiota-immune-nervous system in autism spectrum disorders among students

**DOI:** 10.3389/fmicb.2025.1535455

**Published:** 2025-03-12

**Authors:** Min Zhou, Baoming Niu, Jiarui Ma, Yukang Ge, Yanxin Han, Wenrui Wu, Changwu Yue

**Affiliations:** ^1^Yan’an Key Laboratory of Microbial Drug Innovation and Transformation, School of Basic Medical Sciences, Yan’an University, Yan’an, China; ^2^School of Petroleum Engineering and Environmental Science, Yan’an University, Yan’an, China

**Keywords:** gut microbiota, autism, immune system, nervous system, gut-brain axis

## Abstract

Autism Spectrum Disorder (ASD) is a neurodevelopmental disorder characterized by difficulties in social interaction and communication, repetitive and stereotyped behaviors, restricted interests, and sensory abnormalities. Its etiology is influenced by both genetic and environmental factors, with no definitive cause identified and no specific pharmacological treatments available, posing a significant burden on patients’ families and society. In recent years, research has discovered that gut microbiota dysbiosis plays a crucial role in the pathogenesis of ASD. The gut microbiota can influence brain function and behavior through the gut-brain axis via the nervous system, immune system, and metabolic pathways. On the one hand, specific gut microbes such as *Clostridium* and *Prevotella* species are found to be abnormal in ASD patients, and their metabolic products, like short-chain fatty acids, serotonin, and GABA, are also involved in the pathological process of ASD. On the other hand, ASD patients exhibit immune system dysfunction, with gut immune cells and related cytokines affecting neural activities in the brain. Currently, intervention methods targeting the gut microbiota, such as probiotics, prebiotics, and fecal microbiota transplantation, have shown some potential in improving ASD symptoms. However, more studies are needed to explore their long-term effects and optimal treatment protocols. This paper reviews the mechanisms and interrelationships among gut microbiota, immune system, and nervous system in ASD and discusses the challenges and future directions of existing research, aiming to provide new insights for the prevention and treatment of ASD.

## 1 Introduction

Autism Spectrum Disorder (ASD) is a group of long-term and severe neurodevelopmental disorders characterized by persistent deficits in social communication and restricted (Hyman et al., 2020; Drüsedau et al., 2022), repetitive patterns of behavior, interests, or activities (Fattorusso et al., 2019). In recent years, the global prevalence of autism has continued to rise. According to statistics, the number of people with autism worldwide has already exceeded 67 million (Ha et al., 2021). This indicates that ASD affects many families. The increasing prevalence of ASD over time further highlights that it has evolved into a serious public health issue (Iglesias-Vázquez et al., 2020; Liu et al., 2022), bringing heavy economic and caregiving burdens to society (Hughes et al., 2018).

Although the etiology of ASD has not yet been determined, research reports have indicated that gut microbiota plays a crucial role in its pathogenesis (Erbescu et al., 2022; Chernikova et al., 2021). Abnormal microbes and their induced abnormal metabolism and aberrant development and function of the microbiota-gut-brain axis may be central pathological mechanisms of autism (Sharon et al., 2019). Changes in the gut environment caused by gut microbiota affect the production of signaling substances (Srikantha and Mohajeri, 2019), thereby influencing mature brain function and the development of the central nervous system (CNS) both prenatally and postnatally (Dicks et al., 2021; Doifode et al., 2021; Leblhuber et al., 2021). The gut microbiota-gut-brain axis is the bridge connecting the gut microbiota, the gut, and the central nervous system (Jang et al., 2020). It plays a significant role in various bodily responses, including modulating neuroinflammation, activating the stress axis, enhancing neural transmission, forming the blood–brain barrier, constructing myelin sheaths, maturing microglia, and synthesizing neurotransmitters (Kim and Shin, 2018; Młynarska et al., 2022). Data from preclinical and clinical studies show that this critical axis not only plays a significant role in functional gastrointestinal disorders but also holds substantial potential for new therapeutic targets in a wide range of psychiatric and neurological diseases (Gracie et al., 2019; Person and Keefer, 2021), including Parkinson’s disease, autism spectrum disorders, anxiety, and depression (Martin et al., 2018).

Currently, the treatment options for ASD are minimal (Crowell et al., 2019), with most existing therapies only alleviating surface symptoms and being unable to fundamentally cure the disease (Greenlee et al., 2021). For this reason, it is urgent to deeply explore the causes of ASD and discover new therapeutic targets. Against this background, research focusing on the gut microbiota-immune-neuronal system has brought new hope for tackling the challenges of ASD. This paper will elaborate in detail on the impact of each part of this system on ASD, the mechanisms of interaction among them, as well as the latest treatment advances and future research directions based on these studies, aiming to provide a comprehensive and in-depth theoretical basis and practical reference for the prevention and treatment of ASD.

## 2 Microbiome system and ASD

The human gut harbors trillions of microbes that have co-evolved with their host, weighing approximately 1 kg, making it an integral part of the bod (Adak and Khan, 2019). The collective community of all microbes living in the human gut, such as many different types of known bacteria, viruses, fungi, protozoa, and archaea, is referred to as the gut microbiota (Socała et al., 2021; Halverson and Alagiakrishnan, 2020). Although the gut microbiota is dynamic, it plays important functions in the immunology, metabolism, structure, and microenvironment of the human body (Adak and Khan, 2019).

**Figure 1:**
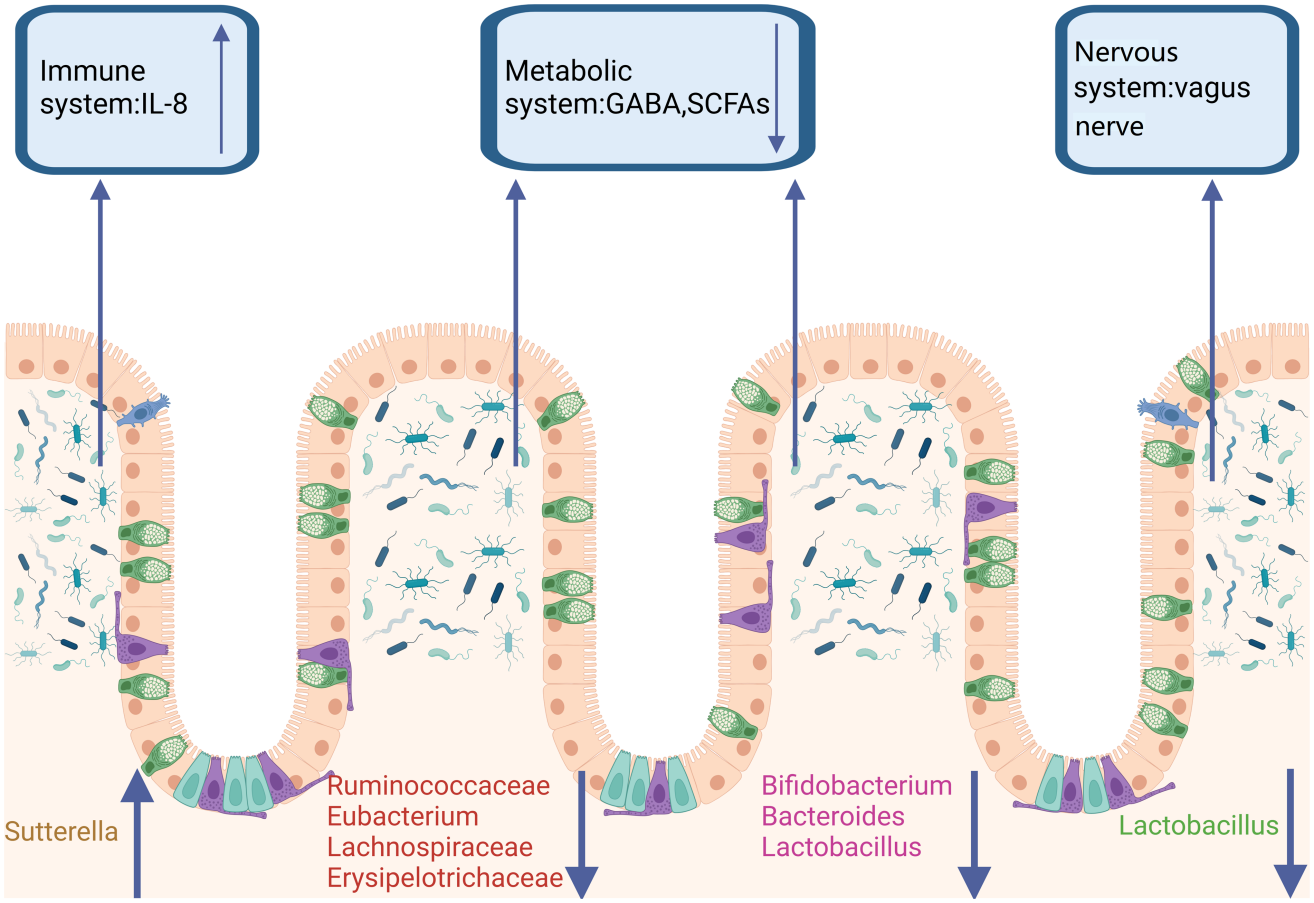
Gut microbiota dysbiosis in ASD. In the feces of ASD patients, a reduction in the abundance of butyrate-producing bacteria, *Ruminococcaceae*, *Bacteroides*, *Lactobacillus*, and *Clostridium* has been observed. It has been reported that in both ASD patients and animal models, the abundance of *Bacteroides*, *Lactobacillus*, and *Bifidobacterium* is decreased, leading to reduced levels of GABA and SCFAs in the metabolic system. Additionally, *Lactobacillus*, which acts through vagal nerve activation, has been shown to improve ASD. *Sutterella*, by reducing mucosal IgA and increasing the pro-inflammatory cytokine IL-8, contributes to ASD through the immune system. Created in https://BioRender.com.

In recent years, as numerous studies have highlighted the bidirectional communication between the gut and the brain, the role of the gut microbiota as a significant factor in the development of ASD has garnered considerable interest (Fattorusso et al., 2019; Góralczyk-Bińkowska et al., 2022). Most studies indicate that alterations in the composition of the gut microbiota in autistic students are linked to gastrointestinal and neurobehavioral symptoms (Fattorusso et al., 2019). The microbes carried by autistic students differ from those of healthy students, particularly in terms of dysbiosis and related intestinal abnormalities (Iglesias-Vázquez et al., 2020). Some abnormal changes in the gut flora affect or even exacerbate symptoms of autistic students through the immune system, nervous system, and metabolic system (Figure 1). A significant number of autism spectrum disorder (ASD) participants exhibit notable gastrointestinal dysfunction, including alterations in bowel habits, chronic abdominal pain, and gastrointestinal symptoms closely associated with the severity of ASD. These symptoms are accompanied by their neurological changes, becoming increasingly severe (Labanski et al., 2020; Sugihara and Kamada, 2021; Gershon and Margolis, 2021).

Currently, researchers have identified several gut microbes associated with autism, including *Clostridium, Prevotella, Coprococcus, Desulfovibrio, Sutterella*, and *Candida albicans* fungi (Chen et al., 2021; Schoeler and Caesar, 2019). These microbes exhibit significant differences between individuals with autism and normal control groups. Additionally, the overall ratios of *Firmicutes* and *Bacteroidetes* also differ from those in normal controls (Fattorusso et al., 2019). Moreover, constipation is a common gastrointestinal issue among ASD patients. Researchers have compared the gut microbiota of individuals with defecation difficulties and those with normal defecation patients (Liu et al., 2022). It has been found that higher levels of *Gemmiger* and *Ruminococcus* in the gut correlate with milder constipation symptoms. In contrast, lower levels of these bacteria correspond to more severe constipation, suggesting a potential protective role for these bacteria (Zhang et al., 2021; Yang et al., 2021, 2022). Furthermore, higher levels of *Escherichia/Shigella* and *Clostridium* clusters XVIII are associated with more severe intestinal symptoms, and these proportions are also elevated in constipated individuals (Dan et al., 2020), indicating that these bacterial groups may act as “disruptors.” Particularly, multiple studies have confirmed an increased presence of *Clostridium* in individuals with autism (Alamoudi et al., 2022; Fu et al., 2021), with *Clostridium* cluster XVIII being capable of producing exotoxins and promoting inflammation (Dan et al., 2020), thus likely contributing to inflammation and the onset of autism. All the above-mentioned are bacteria; there are also differences in fungal composition in the gut between individuals with autism and normal controls (Alamoudi et al., 2022). Candida species are more than twice as prevalent in the gut of individuals with autism compared to healthy individuals. Dysbiosis of gut fungi is associated with the onset of autism, and existing studies have shown that the number of *Candida albicans* significantly increases in individuals with autism (Ding et al., 2021; Zou et al., 2021).

These alterations in gut microbiota influence autism spectrum disorder (ASD) through multiple mechanisms (Paik et al., 2022; Garcia-Gutierrez et al., 2020). Firstly, gut microbiota interacts with the central nervous system via the gut-brain axis, affecting brain function and behavior (Wu and Su, 2024). Neurotransmitters produced by gut microbiota, such as serotonin and dopamine, can influence the brain through the bloodstream or the vagus nerve (Chang et al., 2024). Dysbiosis of gut microbiota may trigger systemic inflammation, and inflammatory factors can cross the blood–brain barrier, impacting brain development and function. Dysbiosis can also activate the immune system, leading to neuroinflammation, which is associated with ASD symptoms. Gut microbiota ferments dietary fiber to produce short-chain fatty acids (SCFAs), such as butyrate and propionate, which can cross the blood–brain barrier and affect brain function (Peralta-Marzal et al., 2021). Specific microbial metabolites, such as ammonia and hydrogen sulfide, may have neurotoxic effects, impairing brain development. Dysbiosis may compromise the intestinal barrier, allowing harmful substances to enter the bloodstream and triggering inflammation and immune responses that affect brain function. Gut microbiota influences gene expression through its metabolites, thereby affecting neurodevelopment and behavior (Gonçalves et al., 2024). In summary, gut microbiota impacts ASD through the gut-brain axis, immune regulation, metabolites, and other mechanisms. A deeper understanding of these mechanisms will aid in the development of new therapeutic approaches.

## 3 The gut-brain axis and ASD

The concept of the “gut-brain axis” was first proposed by Michael Gershon, a neuroscience luminary at Columbia University in the United States. It elucidates the intricate and closely interconnected relationship between the gut, the nervous system (including both the enteric and central nervous systems), and the gut microbiota, collectively constituting the “second brain” within the human body beyond the primary brain (Srikantha and Mohajeri, 2019; Socała et al., 2021; Liang et al., 2018). The gut microbiota influences the brain through the gut-brain axis, which serves as a bidirectional communication bridge between the brain and the intestines. The enteric nervous system, composed of thousands of neurons, regulates gastrointestinal functions, not only transmitting signals but also participating in physiological responses; it maintains internal balance and promotes health (Młynarska et al., 2022; Zhu et al., 2022; Fried et al., 2021).

**Figure 2:**
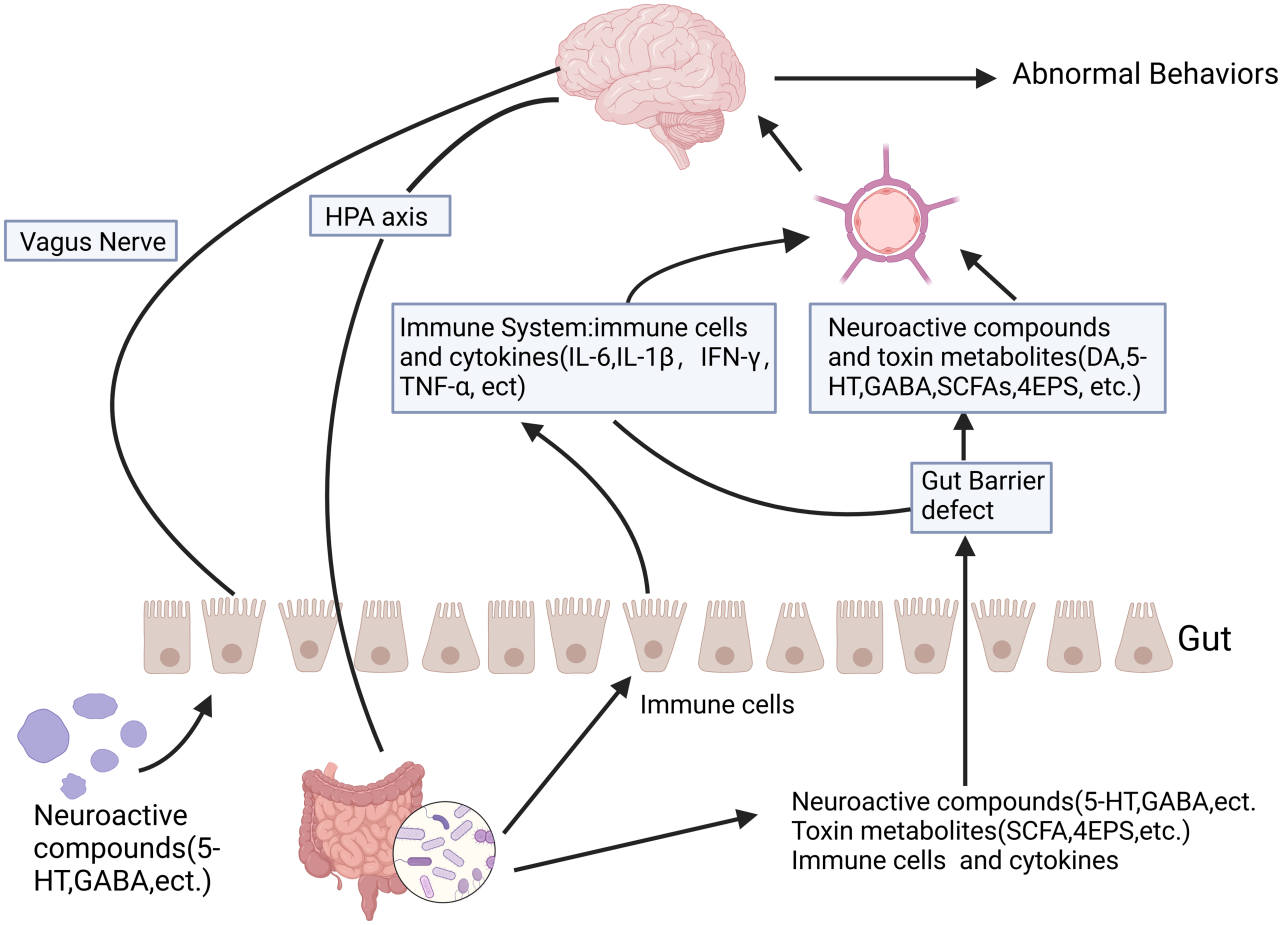
The relationship between autism and the gut microbiota-gut-brain axis is significant. The gut-brain axis facilitates bidirectional communication between the intestines and the brain, involving multiple pathways such as the autonomic nervous system, enteric nervous system, neuroendocrine system, and immune system. Certain metabolic byproducts of microbes, including short-chain fatty acids and toxic metabolites, can influence brain function through the leaky gut pathway. Some microbes produce neuroactive compounds like 5-HT and GABA, which can seep through the leaky gut and affect brain function, potentially leading to abnormal behaviors. These neurochemicals can directly impact the hypothalamic–pituitary–adrenal axis and elevate circulating cortisol levels. Created in https://BioRender.com.

A large body of research currently indicates that there are multiple interaction pathways between the gut microbiota and the brain. The gut microbiota influences brain function through the autonomic nervous system, enteric nervous system, neuroendocrine system, immune system, and toxic metabolites of microbes (Figure 2). Central nervous system disorders caused by an imbalance in the gut microbiota, such as autism, anxiety, depression, and ADHD (Dicks et al., 2021; Sani et al., 2020; Wang et al., 2019), as well as the relationship between heightened brain sensitivity and irritable bowel syndrome, can be considered classic examples of this bidirectional complex relationship (El-Salhy et al., 2019; Vich Vila et al., 2018).

The gut-brain axis is a bidirectional communication system between the gut and the brain, interacting through neural, immune, and metabolic pathways. In recent years, research has shown that gut microbiota plays a significant role in the pathogenesis of autism spectrum disorder (ASD) through the gut-brain axis (Ullah et al., 2023). ASD patients often experience gastrointestinal issues such as constipation, diarrhea, and irritable bowel syndrome, suggesting that gut microbiota dysbiosis may be closely related to the neurobehavioral abnormalities observed in ASD. Firstly, gut microbiota dysbiosis is one of the core mechanisms by which the gut-brain axis influences ASD. The composition of gut microbiota in ASD patients differs significantly from that of healthy individuals, such as abnormal ratios of *Bacteroidetes* to *Firmicutes* (Wiefels et al., 2024). Studies have found that transplanting gut microbiota from ASD patients into germ-free mice can induce ASD-like behaviors. Additionally, gut microbiota influences brain function by producing neurotransmitters (e.g., serotonin, GABA) and modulating immune responses (Li et al., 2021). For example, gut microbiota affects serotonin levels through the tryptophan metabolic pathway, thereby regulating mood and behavior. Secondly, neuroinflammation is another crucial mechanism through which the gut-brain axis impacts ASD. Dysbiosis of gut microbiota can activate the immune system, leading to systemic inflammation and neuroinflammation. Research has shown that ASD model mice exhibit impaired intestinal barrier function accompanied by elevated levels of inflammatory factors (e.g., IL-6, TNF-*α*) (Usui et al., 2023; Zhang et al., 2023). These inflammatory factors can cross the blood–brain barrier, activate microglia, and cause neuroinflammation, thereby affecting neural development and behavior. Metabolites also play a key role in the connection between the gut-brain axis and ASD. Gut microbiota ferments dietary fiber to produce short-chain fatty acids (SCFAs, such as butyrate and propionate), which can cross the blood–brain barrier and influence brain function (He et al., 2023). Studies have demonstrated that injecting propionate into the brains of mice can induce ASD-like behaviors. Furthermore, SCFAs can affect gene expression through epigenetic regulation, thereby influencing neural development and behavior. Impaired intestinal barrier function (i.e., leaky gut) is another critical mechanism by which the gut-brain axis affects ASD. Dysbiosis of gut microbiota may compromise the intestinal barrier, allowing bacterial metabolites and toxins to enter the bloodstream and trigger inflammation and immune responses. Research has found that increased intestinal permeability in ASD patients is associated with elevated levels of inflammatory markers in plasma (Yitik Tonkaz et al., 2023; Piras et al., 2022). In summary, multiple systems influence the pathogenesis of ASD through the gut-brain axis. Future research should focus on exploring this crucial axis in greater depth to better understand the pathological mechanisms of ASD and improve symptoms in ASD patients.

## 4 Nervous system pathways and ASD

Neurological pathways play a central role in the pathogenesis of ASD, involving multiple aspects such as neuronal development, synaptic function, neurotransmitter systems, neuroinflammation, and epigenetic regulation. The nerve pathway is a direct channel connecting the gut and the brain, mainly consisting of the vagus nerve and the enteric nervous system (ENS). Among them, the vagus nerve originates from the brainstem. It is responsible for regulating the functions of visceral organs, making it one of the fastest and most direct paths for the gut microbiota to influence the brain (Patrono et al., 2021; Craig et al., 2022; Fülling et al., 2019). The vagus nerve is a pair of nerves composed of afferent and efferent neurons, allowing bidirectional information transfer between the brain and the gut. Some microbiota have been demonstrated to interact with the brain and modulate central nervous system (CNS) functions and related behaviors (Vendrik et al., 2020; Vicentini et al., 2021; Panther et al., 2022). For instance, improvements in anxiety-related and depressive behaviors by gut microbiota can be blocked by vagotomy (Panther et al., 2022; Liu et al., 2020), and modulation of behaviors related to autism and oxytocin signaling pathways has also been shown to be dependent on the vagus nerve (Israelyan and Margolis, 2018). The neural pathway, particularly the vagus nerve pathway, has been well established as mediating the connection between the gut microbiota and the central nervous system. In various ASD models, vagotomy has demonstrated that the vagus nerve plays a crucial role in the interaction between the gut microbiota and the brain (Vich Vila et al., 2018; Yu and Zhao, 2021).

Neurotransmitters are chemical substances that transmit signals between neurons, and individuals with autism spectrum disorder (ASD) often exhibit abnormalities in multiple neurotransmitter systems. Research has shown that while levels of serotonin (5-HT) are elevated in the blood of ASD patients, its functionality in the brain may be reduced, affecting emotion and behavior regulation (Dan et al., 2020). Additionally, gamma-aminobutyric acid (GABA), the primary inhibitory neurotransmitter, may have impaired signaling in ASD patients, leading to an imbalance between excitation and inhibition. Glutamate, the primary excitatory neurotransmitter, also shows abnormal signaling, which may be associated with cognitive and behavioral impairments in ASD (Saha et al., 2022). A review has pointed out that abnormalities in the 5-HT and GABA systems in ASD patients may be linked to social and cognitive deficits. At the same time, other studies have found that the imbalance between excitation and inhibition is a key pathological mechanism in ASD. Neuroinflammation, another significant pathological feature of ASD, may affect neuronal function by activating microglia and releasing inflammatory factors. This is often observed in the brains of ASD patients, where activated microglia release inflammatory factors that can damage neuronal and synaptic function. Furthermore, elevated levels of inflammatory factors (such as IL-6 and TNF-*α*) in the blood and cerebrospinal fluid of ASD patients may cross the blood–brain barrier and impact brain function (Megagiannis et al., 2024). Studies have already identified significant upregulation of genes related to immunity and inflammation in the brains of ASD patients. Epigenetic mechanisms, such as DNA methylation and histone modification, also play a crucial role in the pathogenesis of ASD, potentially influencing neurodevelopment by regulating gene expression (Smith et al., 2020). Epigenetic changes may affect the expression of genes related to neuronal development and synaptic function. Environmental factors, such as maternal infection or toxin exposure, may further influence the risk of ASD through epigenetic mechanisms (Yenkoyan et al., 2024). Research has found abnormal expression of genes associated with epigenetic regulation in the brains of ASD patients. These abnormalities in neurotransmitter systems, neuroinflammation, and epigenetic regulation may collectively contribute to the brain dysfunction and behavioral impairments observed in ASD patients. Future research should further explore the specific molecular pathways underlying these mechanisms and develop intervention strategies targeting neurological pathways to improve ASD symptoms.

## 5 Immune system pathways and ASD

The gut is the largest immune organ in the human body, with 70% of the immune system located in the gut (Wiertsema et al., 2021). The gut microbiota has been shown to regulate the intestinal mucosal immune system, the systemic immune system, and the function of central nervous system immune cells, thereby directly or indirectly regulating neural activity (García-Montero et al., 2021; Michaudel and Sokol, 2020).

A substantial amount of evidence indicates persistent immune dysregulation in ASD patients. A substantial body of evidence indicates that the immune system of individuals with Autism Spectrum Disorder (ASD) exhibits persistent dysregulation. Early studies on the immunopathology of ASD revealed a reduced lymphocyte response to mitogen stimulation in ASD students, suggesting that lymphocyte subsets play a role in the pathological process of ASD. In recent years, an increasing number of studies have further confirmed the significance of cellular immune responses in autism. Studies by Ahmad et al. (2019a) suggest that IL-16 expression may play a significant role in abnormal immune responses in ASD patients. Compared to typically developing (TD) controls, ASD children exhibited significant increases in CD4IL-16, CD8IL-16, CD14IL-16, CCR3IL-16, and CXCR7IL-16 cells, along with elevated expression of IL-1βIL-16, IL-6IL-16, and TNF-αIL-16. Additionally, compared to TD controls, IL-16 mRNA and protein expression were significantly induced in ASD children (Ahmad et al., 2019a). Ki-67 plays a crucial role in many neurological diseases, and studies have shown disrupted Ki-67 expression in T cells from ASD children. Specifically, there was a significant increase in CD3+Ki-67+, CD4+Ki-67+, CD8+Ki-67+, CXCR4+Ki-67+, CXCR7+Ki-67+, CD45R+Ki-67+, HLA-DR+Ki-67+, CXCR4+GATA3+, and GATA3+Ki-67+ cells, as well as Helios+Ki-67+ and FOXP3+Ki-67+ cells. Moreover, compared to TD controls, ASD children showed upregulated Ki-67 mRNA levels (Alhosaini et al., 2021).

A recent meta-analysis indicated significant reductions in CD4+ lymphocytes in ASD patients, with an increase in CD4+ memory T cells associated with HLA A2-DR11 and a decrease in CD4+ naive T cells, as well as an imbalance in cytokine production by CD4+ and CD8+ T cells. Notably, the rate of Tregs in the peripheral blood of ASD patients was lower, while the percentage of Th17 increased, leading to a marked shift in the Th17/Treg balance (Ellul et al., 2021; Chua et al., 2020). This is attributed to a decreased ratio of IFN-*γ* and IL-2 produced by CD4+ and CD8+ T cells. Regulatory T cells (Tregs) play a key role in regulating immune responses. Research by Sherlly Xie et al. reported fewer CD4+CD25high Tregs in the blood of children with autism and also reported dysregulation of related transcription factors for Th1, Th2, Th17, and Tregs (Chua et al., 2020; Xie et al., 2019).

T lymphocyte dysregulation has been observed in numerous ASD patients, primarily characterized by abnormal T helper-suppressor cell ratios, systemic defects in regulatory T cells, and dysregulated cytokine release in individuals with autism (Moaaz et al., 2019). Therefore, the Th-17 lymphocyte subset plays a significant role in ASD immunopathology, with Th17 cells playing a central role in the pathogenesis of ASD through the release of IL-17A (Ahmad et al., 2018; Ahmad et al., 2019b). Nadeem et al. (2020) studied IL-6R/sIL-6R and IL-17A-related markers (p-STAT3, IL-17A, IL-23R) in the peripheral blood of ASD children and TD controls, demonstrating that overactive IL6 signaling may be associated with upregulated differentiation or development of Th17 cells in ASD subjects. Thus, treatment strategies targeting IL-6 or IL-17A-related signal transduction may be beneficial for ASD.

Microglia are the macrophages of the central nervous system, responsible for maintaining neural networks and repairing damage. The gut microbiota influences them, and this regulatory effect occurs in a time- and sex-dependent manner (Doroszkiewicz et al., 2021). Additionally, the microbial metabolites of tryptophan can modulate inflammation in the central nervous system by activating the aryl hydrocarbon receptor on astrocytes. The gut microbiota also communicates with the brain through interactions with intestinal and peripheral immune cells (Yu and Zhao, 2021).

Intestinal immune cells exert an impact on brain neuroactivity through both direct and indirect mechanisms (Dicks et al., 2021). In specific circumstances, such as during brain injury, these cells can regulate neural activity in the brain either by directly crossing the blood–brain barrier (BBB) or by indirectly connecting via afferent fibers and enteric nerves. Specific cytokines are able to pass directly through the BBB (Rutsch et al., 2020), modifying the inflammatory state of the central nervous system. For example, increased levels of IL-6 in the brain can disrupt the balance between excitatory and inhibitory synaptic transmission, resulting in behaviors associated with autism spectrum disorder (ASD). It is important to note that the gut microbiota may influence the development and integrity of the BBB. Moreover, impairment of the BBB’s integrity enhances the brain’s susceptibility to pathogens and microbial products circulating in the system (Ahmad et al., 2019a; Majerczyk et al., 2022).

## 6 Metabolic pathways and ASD

Chemical molecules produced by the gut microbiota can penetrate the intestinal epithelial barrier and cross the blood–brain barrier, directly influencing the brain. Additionally, these chemical molecules can indirectly transmit signals through interactions with enteroendocrine cells scattered throughout the intestinal epithelium (Feng et al., 2024). The gut microbiota primarily utilizes three types of signaling molecules to directly or indirectly regulate the homeostasis of the central nervous system: firstly, food-related metabolites such as short-chain fatty acids (SCFAs); secondly, endogenous molecule metabolites like tryptophan metabolite serotonin (5-hydroxytryptamine [5-HT]), bile acids, and gamma-aminobutyric acid (GABA); thirdly, components of the microbial cell wall, such as lipopolysaccharides.

### 6.1 Short-chain fatty acids (SCFAs)

Short-chain fatty acids (SCFAs) primarily include acetate, propionate, and butyrate. These substances are either excreted or absorbed in the colon. Among them, butyrate is an essential fuel for colon cells and has anticarcinogenic and anti-inflammatory properties. The ratio of these fatty acids depends on the types of gut bacteria and dietary structure. Clostridium can produce a large amount of propionate, which, when entering the bloodstream, can be toxic to the brain and may affect the emotions and behavior of children with autism spectrum disorder (ASD) (Liu et al., 2019). The gut microbiota produces short-chain fatty acids (SCFAs) through the fermentation of dietary fiber in the intestine. SCFAs can cross the blood–brain barrier and directly affect neural processes. In the brain, SCFAs act as endogenous ligands for extracellular G protein-coupled receptors and regulate gene expression intracellularly by inhibiting histone deacetylase. However, due to the small amount of SCFAs absorbed by the brain, an increasing number of studies suggest that SCFAs influence brain function through indirect pathways (Tran and Mohajeri, 2021). SCFAs can also affect the microbiota-gut-brain axis through neuroimmune pathways by reducing systemic inflammation and modulating the activation of microglia associated with neuroinflammation. SCFAs alleviate systemic inflammation primarily through two mechanisms. First, SCFAs can enhance the function of the intestinal epithelial barrier, reducing the entry of pathogens and metabolic products into the peripheral blood. Second, SCFAs can enter the peripheral blood and directly interact with various immune cells, thereby improving systemic inflammation. Finally, SCFAs may mediate communication between the microbiota-gut-brain axis by regulating gut endocrine pathways and the vagus nerve pathway (Deng et al., 2022).

### 6.2 Serotonin (5-HT)

5-Hydroxytryptamine (5-HT), also known as serotonin, is a widely distributed monoamine neurotransmitter in the human body that plays a crucial role in brain development by regulating various developmental processes such as neuronal migration, cell differentiation, and synaptogenesis. Some of these events are associated with autism spectrum disorder (ASD). In the gut, 5-HT is produced from tryptophan by enterochromaffin cells, a process regulated by the gut microbiota. Studies have found that ASD patients often exhibit increased levels of 5-HT in peripheral blood but decreased levels in the brain. Additionally, abnormalities in the serotonin transporter (5-HTT) gene have been observed. Serotonin in the brain is also responsible for social responses; research indicates that a deficiency in the 5-HT gene can lead to decreased social abilities, while the release of 5-HT in the nucleus accumbens can improve social deficits (Walsh et al., 2021; Walsh et al., 2018).

### 6.3 Gamma-aminobutyric acid (GABA)

*γ*-Aminobutyric acid (GABA), as a major inhibitory neurotransmitter, is produced not only by gut microbes such as *Bacteroides*, *Lactobacillus*, *Bifidobacterium*, and *Parabacteroides* but also significantly influenced by the environmental pH. Among these microorganisms, Bacteroides demonstrates the most substantial potential for GABA production within the specific pH range of the human colon. Furthermore, gut-derived GABA participates in the intricate gut-brain connection through both endocrine and neuroimmunological pathways. Notably, the presence of GABA transporters on brain capillary endothelial cells suggests that GABA may cross the blood–brain barrier to directly exert its inhibitory neurotransmitter function within the central nervous system (Dan et al., 2020; Maier et al., 2022).

### 6.4 Lipopolysaccharides (LPS)

Lipopolysaccharides (LPS) are essential components of the outer membrane of certain bacteria. Under normal circumstances, they are blocked from entering the bloodstream by the tight junctions of intestinal wall cells. However, when there is a disturbance in the gut microbiota and permeability increases, lipopolysaccharides can enter the bloodstream, cross the blood–brain barrier, and launch an attack on the brain with pro-inflammatory chemicals, triggering a series of behaviors such as autism. Li et al. (2023) used dextran sulfate sodium (DSS) to disrupt the intestinal barrier of BTBR T + tf/J autistic mice and metformin to repair the intestinal barrier to test this hypothesis. The results showed that DSS treatment led to a reduction in social approach behavior. Still, after taking metformin, the autistic behaviors of the mice were improved, social memory affinity increased, and repetitive and anxiety-related behaviors decreased. After taking metformin, the concentration of lipopolysaccharides in the blood decreased. Therefore, “Leaky gut” could be a crucial factor in the onset of autism through the stimulation of the lipopolysaccharide-induced TLR4-MyD88-NF-κB pathway.

### 6.5 4-Ethylphenolsulfate (4EPS)

**Figure 3:**
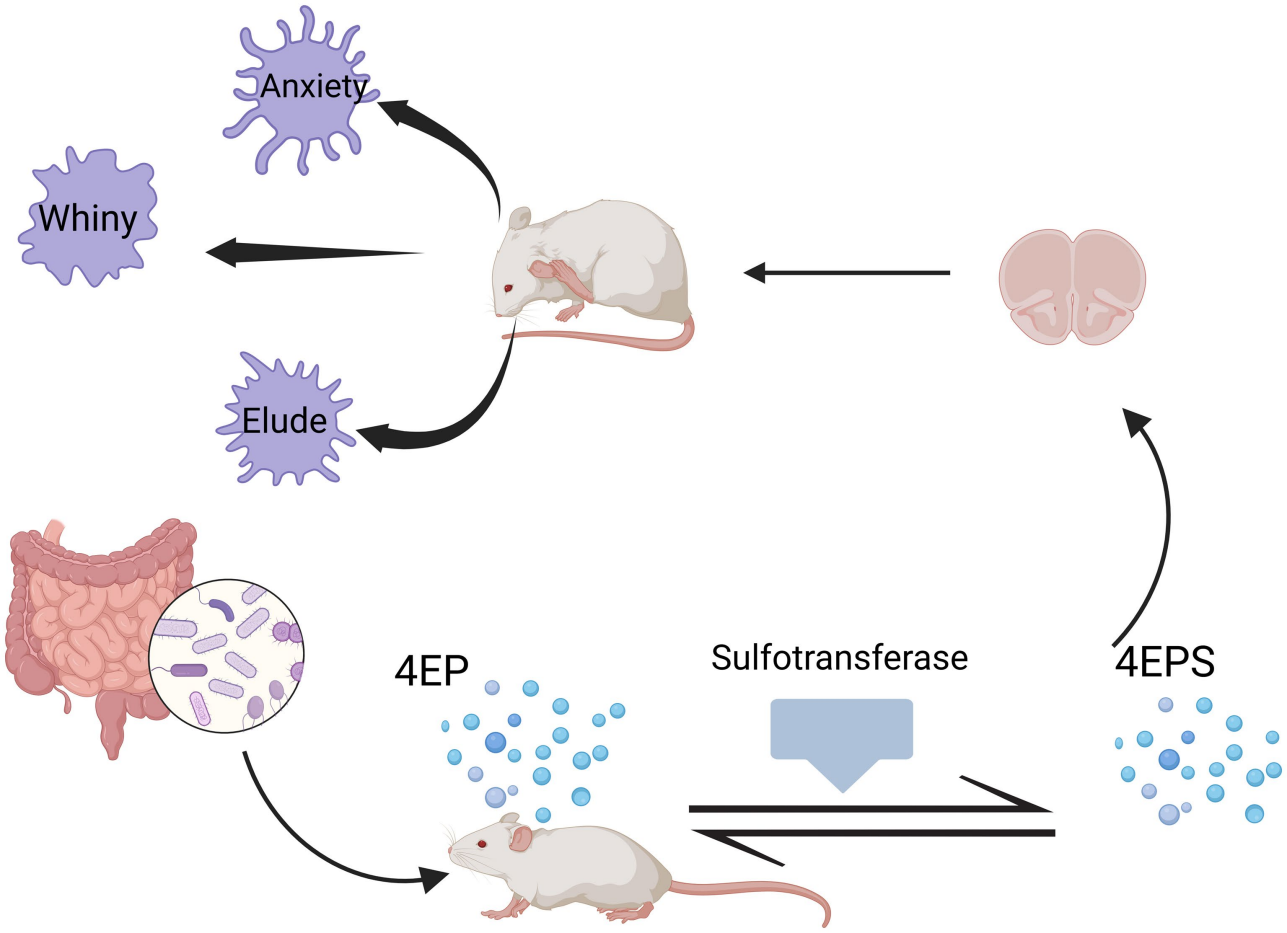
Production of 4EPS and its effects on mice. Different species of *Clostridium* within the gut microbiota can metabolize tyrosine or other dietary sources to produce 4EP, which is then converted into 4EPS in the mouse body by sulfotransferases. Subsequently, it enters the brain, altering the activation and connectivity of specific brain regions, and disrupting the maturation of oligodendrocytes and myelin formation processes, can induce a variety of emotional behaviors in mice. Created in https://BioRender.com.

4-Ethylphenolsulfate (4EPS) is a neurotoxin. Needham et al. (2022) identified various species of *Clostridium* from the gut microbiota that can metabolize tyrosine or other dietary sources to produce 4EP, which is then converted into 4EPS in the host by sulfotransferases. Subsequently, it enters the brain, altering the activation and connectivity of specific brain regions, and disrupting the maturation of oligodendrocytes and myelin formation processes, and can induce a variety of emotional behaviors in mice (Figure 3).

## 7 Other factors and ASD

Genetic factors occupy a central position in the occurrence of autism. As an essential component of the human body, the composition and function of the gut microbiota are also profoundly influenced by genetic factors (Masini et al., 2020). Studies have shown that an individual’s genetic background can shape their unique gut microbiota structure, and these structural differences may be related to the risk of autism (Antaki et al., 2022). Specific gene variations may interfere with the normal function of the intestinal barrier, affect immune response mechanisms, or alter metabolic pathways, thereby indirectly disrupting the balance of the gut microbiota. At the same time, the gut microbiota may also interact with host genes by regulating gene expression and producing metabolic products, and together they affect the developmental process of autism. In addition, prenatal stress, including multiple aspects of psychological, physiological, and environmental stress experienced by the mother during pregnancy, has a long-term impact on the construction of the fetal gut microbiota. These stress factors may lead to changes in maternal hormone levels, which in turn affect the development of the fetal gut and the colonization of the microbiota (Cirnigliaro et al., 2023; Wei et al., 2021). Research has shown that prenatal stress is associated with reduced diversity of the infant gut microbiota after birth, imbalances in specific bacterial species, and other abnormalities (Woo et al., 2023). These abnormalities may further affect the infant’s immune development, metabolic functions, and neurobehavioral manifestations, thereby increasing the risk of neurodevelopmental disorders such as autism (Beversdorf et al., 2018). Besides, environmental exposure is also one of the critical factors affecting the gut microbiota. Factors including, pollutants, drugs, and diet can significantly impact the gut microbiota. Pesticides, heavy metals, and other environmental contaminants can enter the human body through the food chain, disrupt the balance of the gut microbiota, induce inflammation, and lead to immune dysfunction (Awadh et al., 2023). The widespread use of antibiotics is another major cause of gut microbiota imbalance. While antibiotics eliminate harmful bacteria, they may also disrupt the balance of beneficial bacteria, thereby increasing the risk of health problems such as autism (Ahrens et al., 2024).

In summary, genetic background, prenatal stress, and environmental exposure collectively constitute key factors shaping the gut microbiota, and these factors are closely related to the development of autism. Therefore, when exploring the relationship between autism and the gut microbiota, we need to comprehensively consider the interactions of these factors. Future research should further reveal the internal connections and mechanisms of these factors, providing new ideas and methods for the prevention and treatment of autism. For example, adjusting the flora structure through probiotic or prebiotic supplementation is also expected to become a significant innovative direction in the field of autism treatment. The implementation of these strategies may not only directly improve the health status of the gut microbiota but also positively influence the symptoms of autism through pathways such as regulating immune responses, metabolic processes, and neurobehavioral manifestations.

## 8 Probiotics and prebiotics

Probiotics primarily refer to a class of live microorganisms that, when ingested in sufficient quantities, can confer beneficial effects on the host organism. They inhibit the proliferation of harmful bacteria, aid in nutrient absorption, and safeguard intestinal health. Common types include genera such as *Bifidobacterium*, *Lactobacillus*, *Enterococcus*, and *Saccharomyces* (Wieërs et al., 2019). Prebiotics are a group of non-digestible compounds that enhance the growth and function of probiotics while suppressing the expansion of detrimental bacteria (Simon et al., 2021). Research indicates that patients, especially the infant stage, are a critical period for establishing a regular microbial community. A well-established microbiota can resist pathogen invasion, create a favorable nutritional environment, boost immunity, and prevent and treat diseases (Crowell et al., 2019; Girault and Piven, 2020). Although research is limited, some studies (Table 1) suggest that *Lactobacillus plantarum* significantly alleviates stress and anxiety and improves cognitive function, potentially enhancing the mental focus of children with autism. *Bifidobacterium* has been clinically validated for its intervention effects in various diseases, particularly in digestion, allergies, and cognition. *Lactobacillus reuteri* can intervene in the synthesis and secretion of neurotransmitters, influence the vagus nerve system, relieve anxiety, enhance cognition, and improve social behavior deficits; it has also been proven to aid in the intestinal health of students with IBD, which is common among students with autism (Duque et al., 2021; Arnold et al., 2019).

**Table 1:** Probiotics improve ASD symptoms.

Probiotics	Effect	Mechanism	References
*Lactobacillus plantarum*	Significantly alleviates stress and anxiety, improves cognitive abilities	Improves ASD by activating the vagus nerve	Chen et al. (2024)
*Bifidobacterium*	Improves digestion, allergies, and cognition in ASD patients	The Firmicutes phylum significantly decreased, while the ratio of Bacteroidetes to Firmicutes was restored. The relative abundance of Lactobacillus bacteria significantly increased, and gastrointestinal symptoms were notably alleviated.”	Alsubaiei et al. (2023)
*Lactobacillus GG (LGG)*	Constipation symptoms were significantly improved, with increased frequency of bowel movements, reduced stool hardness, lower frequency of fecal incontinence, and decreased abdominal pain.	Increase beneficial bacteria in the ASD gut, reduce harmful bacteria such as Clostridia, and restore SCFAs levels	Nettleton et al. (2021)
*Lactobacillus reuteri*	Alleviates anxiety, improves cognition, and ameliorates social behavior deficits	Interferes with the synthesis and secretion of neurotransmitters, affecting the vagus nerve system	Zeng et al. (2024)
*Clostridium scindens*	Intestinal permeability and autism-like behaviors were improved	The ratio of Bacteroidetes to Firmicutes, as well as the proportions of Desulfovibrio and Bifidobacterium bacteria, were all restored	Morton et al. (2023)

Modulating the gut microbiome represents a promising approach to decrease the prevalence of detrimental microorganisms and their associated byproducts that adversely affect behavior regulated by the central nervous system (CNS), or to enhance the presence of beneficial microbes and their metabolites that bolster normal CNS operations. As a result, probiotics and prebiotics are increasingly recognized as gentle, alternative therapeutic options for neurological conditions such as Autism Spectrum Disorder (ASD) (Abdellatif et al., 2020). Research has indicated that probiotics and prebiotics can alleviate hopelessness and anxiety-like symptoms in mice subjected to chronic stress (Marin et al., 2017). Investigations conducted by Sgritta et al. (2019) revealed that probiotic intervention could correct social impairments across various autism mouse models. Furthermore, *Bifidobacterium longum*, a widely endorsed probiotic, has been demonstrated to avert autoimmune disorders like inflammatory bowel disease and restore normalcy to anxiety-like behavior in mice suffering from persistent intestinal inflammation (Zhang et al., 2017; Abujamel et al., 2022). Oral administration of *B. longum* to rats under “stressful” circumstances suggested that this therapy influenced the activity of the hypothalamic–pituitary–adrenal pathway and mitigated anxious behavior (Haas et al., 2020).

Regulating the gut microbiota is a potential strategy to reduce the abundance of harmful microbes and related metabolites that negatively impact central nervous system (CNS)-driven behaviors or increase the abundance of microbes and metabolites that support normal CNS-driven behaviors. Therefore, probiotics and prebiotics are becoming non-invasive treatment alternatives for neurological disorders, including ASD. Probiotics and prebiotics have improved despair and anxiety-like behaviors in chronically stressed mice. Sgritta et al. (2019) used probiotic therapy to reverse social deficits in several autism mouse models. Additionally, *B. longum* is a well-evidenced probiotic that has been shown to prevent autoimmune diseases like inflammatory bowel disease and normalize anxiety-like behaviors in mice caused by chronic intestinal inflammation. Oral administration of *B. longum* to rats under “stress” conditions indicated that this treatment affected hypothalamic–pituitary–adrenal axis function and reduced anxiety behaviors.

In summary, these findings highlight the potential of administering probiotics and prebiotics as a microbiome-targeted therapy, which may effectively improve ASD-related symptoms by modulating the gut microbiota.

## 9 Fecal microbiota transplantation (FMT)

Fecal microbiota transplantation (FMT) is an emerging therapeutic approach that involves introducing functional microbial communities from the feces of healthy donors into patients to treat a variety of diseases, including infections, immune disorders, liver diseases, enteric brain disease, and cancer (Ooijevaar et al., 2019). Unlike probiotics or other treatments, FMT directly targets the gut microbiome and is considered a safe and effective method for re-establishing intestinal microecology (Li et al., 2022; Avolio et al., 2022). Studies have shown that FMT has good tolerance and effectiveness in improving gastrointestinal symptoms and autism-related behaviors in patients with Autism Spectrum Disorder (ASD). FMT can induce a new microbial community that significantly differs from the pre-FMT community and is more similar to the microbial communities of healthy donors and typically developing children (Li et al., 2021). Research reports indicate that children who received FMT showed significant reductions in scores on the Childhood Autism Rating Scale (CARS) (Chen and Chiu, 2022). Additionally, the study found that FMT therapy reduced the relative abundance of *Bacteroides fragilis* and continuously shifted the gut microbiota of autistic individuals toward a healthier state. The research by Kang et al. (2019), further demonstrated that after fecal microbiota transplantation, overall microbial diversity increased, and the abundance of *Bifidobacterium*, *Prevotella*, and *Desulfovibrio* also increased, with these changes persisting after treatment.

Bacterial therapies based on altering the gut microbiome composition through fecal microbiota transplantation may be a promising approach for treating gastrointestinal disorders and behavioral traits associated with ASD (Żebrowska et al., 2021). However, further research is necessary due to the limited long-term efficacy data of this therapy. It can be anticipated that, based on current scientific evidence and experimental studies, these therapies will be developed in the future to treat gastrointestinal diseases related to ASD, which may also have secondary beneficial effects on behavioral symptoms (Suprunowicz et al., 2024). Observing the research direction in neurological diseases, the FMT method seems poised to become an important area of study in the coming years (Vendrik et al., 2020).

## 10 Discussion

This study focuses on the relationship between gut microbiota and autism spectrum disorder (ASD), revealing potential connections among microbiota, the immune system, and the nervous system. However, there are several limitations in the current research. The sample size is relatively small and primarily concentrated on a specific population, at the same time, significant differences exist in the structure and function of gut microbiota among different populations, limiting the generalizability of the findings. The research methods also lack standardization; for example, diverse techniques such as 16S rRNA sequencing and metagenomic sequencing are used to detect gut microbiota, and the varying results produced by different methods affect comparability. Additionally, the high variability of gut microbiota increases the complexity of the study, reduces the reproducibility of results, and complicates statistical analysis. In terms of interventions, the long-term safety of fecal microbiota transplantation (FMT) and probiotics has not been thoroughly explored, potentially involving risks such as the transfer of pathogenic microorganisms or undesired phenotypes. Furthermore, the pathogenesis of ASD is complex, involving genetic, environmental, and immune factors, among others, with gut microbiota being only one component. Current studies often struggle to comprehensively consider the interactions among these factors, limiting the overall understanding of ASD mechanisms.

Anxiety and depression are highly prevalent among students with ASD. These comorbid conditions can cause additional impairments, necessitating targeted treatments (White et al., 2018). Future research should expand sample sizes, establish standardized methods, consider multifactorial influences, and provide more detailed data interpretation to enhance the scientific and practical value of the studies. For example, linking changes in microbiota composition and metabolite profiles at specific time points to variations in ASD symptoms resulting from different types of treatments could be insightful. Two critical areas for future research include the gender differences in gut microbiota composition and a deeper understanding of the impact of early-life stress and inflammation on gut microbiota. Additionally, further exploration of the effects of gender differences, early-life stress, and inflammation on gut microbiota is needed to provide a more comprehensive basis for individualized ASD treatments (Horecka-Lewitowicz et al., 2024).

Although the use of probiotics and prebiotics has garnered attention, future research should focus more on FMT, a technique for repopulating gut microbiota. Current cutting-edge research also suggests other potential therapeutic approaches that may benefit individuals with ASD, including: AB-2004 (also known as cilansetron, an edible activated carbon particle that non-selectively adsorbs harmful microbial metabolites in the gut), food extracts such as anthocyanins, and non-invasive brain stimulation techniques like transcranial magnetic stimulation (TMS) and transcranial direct current stimulation (tDCS), which modulate cortical activity and improve social and behavioral symptoms in ASD patients (Gerges et al., 2024). However, these studies are still limited to small participant groups or animal trials, and large-scale applications will require more time (Palanivelu et al., 2024).

In conclusion, developing individualized treatment plans for each student with ASD requires clinicians to conduct comprehensive, detailed, and rigorous assessments to select the most beneficial integrated treatment approach for maximum patient benefit.

## 11 Conclusion

Despite certain debates and lingering questions about the integration and composition of the gut microbiome, the gut-brain axis holds paramount significance for advancing our understanding of the origins and therapeutic approaches to ASD. Incorporating the microbiome into clinical psychiatry is vital for pinpointing biomarkers associated with biological diversity, thereby improving the capacity to align patients with their most suitable treatments; identifying at-risk individuals eligible for prompt intervention; uncovering novel targets for pharmaceutical innovation; and formulating treatments that target the microbiome, such as dietary modifications, probiotic supplementation, and prebiotic administration. In addition, the long-term safety of treatments such as FMT (fecal microbiota transplantation) and probiotics has not yet been thoroughly explored. Future research should delve deeper into the specific molecular mechanisms by which gut microbiota influences ASD through the gut-brain axis, including neurotransmitters, metabolites, and immune regulation. By integrating multi-level research methods and technological approaches, it is possible to unravel the complex mechanisms of ASD and develop more effective diagnostic and therapeutic strategies, ultimately improving the quality of life for individuals with ASD.
